# Body mass index mediates the association between sleep duration and academic performance: An evidence in Chinese adolescent students

**DOI:** 10.1371/journal.pone.0323969

**Published:** 2025-06-05

**Authors:** Linni Gu, Rui Zhu, Donghua Tian

**Affiliations:** 1 Health Management School, Inner Mongolia Medical University, Huhehaote City, China; 2 China Academy of Social Management, Beijing Normal University, Beijing, China; 3 School of Sociology, Beijing Normal University, Beijing, China; University of Health and Allied Sciences, GHANA

## Abstract

**Background:**

Academic performance serves as a crucial indicator for evaluating adolescents’ educational outcomes in China, which prompting parents and schools to place significant emphasis on students’ academic achievements. However, the pursuit of excellent academic performance often leads to inadequate sleep duration, while the body mass index (BMI) is also disregarded when prioritizing academic achievement. This study aims to examine the association between sleep duration and academic performance and to explore the mediating role of body mass index (BMI) among adolescent students in rural and urban areas of China.

**Methods:**

The study utilized baseline (2013–2014 academic year) and follow-up survey data (2014–2015 academic year) from the China Education Panel Study (CEPS). A total of 7,218 adolescent subjects aged between 12 and 14 were recruited using multi-stage random probability proportional to size sample (PPS) sampling, providing demographic, sleep duration, and body mass index (BMI) information. Descriptive analysis, Ordinary Least Square (OLS), and Product of Coefficients Approach were used to estimate the association and mediating role between sleep duration, BMI and academic performance.

**Results:**

The sleep duration has significantly influenced the academic performance of adolescent students, (*p* < 0.01). A 10-minute increase in sleep duration was associated with a 6.3% decrease in academic performance for rural-urban students (*β* = -0.063, *p* < 0.01). Subgroup analyses further demonstrated that a 10-minute increase in sleep duration led to an 8.5% decrease in academic performance for rural adolescent students (*β* = -0.085, *p* < 0.01), while urban students experienced a 6.7% decrease under similar circumstances (*β* = -0.067, *p* < 0.05). Body mass index (BMI) negatively influenced students’ academic performance (*β* = -0.071, *p* < 0.01) and mediates the relationship between sleep duration and academic performance (*β* = 0.112, *p* < 0.001).

**Conclusions:**

The sleep duration has negatively influenced rural-urban adolescent students’ academic performance in China, however, this influence was indirect when added the variable BMI into the model. The BMI played a mediating role in the relationship between sleep duration and academic performance.

## Background

The academic performance of adolescents in China has been a significant concern by parents and societies, as it serves as a crucial indicator for evaluating their educational achievement and potentially influencing their future career choices [1]. Consequently, the sleep duration of adolescent students has been reduced to allocate more time for studying [2]. Recent statistics from the China Youth and Children Research Center indicate that nearly 60% of Chinese students have encountered sleep insufficiency in the past decade [2]. Specifically, elementary school students on average have been found to sleep less than nine hours [2,3], while over 57% of adolescent students sleep less than eight hours on study days during the week, with over 34.5% sleeping less than nine hours on weekends [2,3].

This phenomenon has generated significant attention from researchers. Numerous studies have investigated the association between sleep and students’ academic performance [4–8]. A preliminary study has demonstrated that sleep deprivation leads to poor leaning outcomes among students [9]. Another similar study has highlighted the detrimental effects of inadequate sleep on cognitive development of students, resulting in lower academic performance among higher education students [10]. Conversely, a recent study has provided an opposing conclusion by demonstrating that excessive sleep duration exceeding 10 hours can impede academic performance [11].

Meanwhile, other studies have explored the significant of physical health on students’ academic performance [11]. The body mass index (BMI) has been identified as a crucial factor for assessing students’ physical health [12,13]. A preliminary study has revealed that obese adolescent students demonstrated lower academic achievement compared to their non-obese counterparts [14]. Furthermore, recent research identified a negative correlation between overweight and students’ academic performance [15]. Similarly, a study conducted in China testified that underweight also had an negatively influenced on students’ academic performance [16]. A plethora of research have consistently confirmed that overweight and obesity in adolescents were associated with cardiovascular disease, metabolic complications, and chronic illnesses [17–19], which ultimately affect mental development, intelligence growth, consequently influencing their academic performance [20]. Additionally, previous finding illustrated that underweight was linked to malnutrition leading to cerebral agenesis [21].

Overall, the present studies have explored the association between sleep and students’ academic performance from various aspects. The inconsistent results explained that sleep duration plays a crucial role in influencing students’ academic performance. However, no previous studies have examined the association between sleep duration, body mass index (BMI) and academic performance in adolescent students. Therefore, the primary objective of this study is to testify whether sleep duration positively or negatively influences academic performance in adolescent students and also investigate potential variations in this influence between urban and rural students. The second objective is to elucidate the mediating role of BMI between sleep duration and academic performance in rural-urban adolescent students. Therefore, we propose the hypothesis that sleep duration influences academic performance indirectly through its association with BMI.

## Methods

### Data source and sampling

The data for this study was obtained from the China Education Panel Survey (CEPS), which was conducted by the China Survey and Data Center of Renmin University of China. CEPS provides a comprehensive investigation on various aspects of Chinese society, ensuring a robust representation of the nation’s situation. The baseline study was conducted during the academic year from 2013 to 2014, with subsequent follow-up research conducted in academic year 2014–2015 under the supervision of the Data Center. To ensure the representativeness, a multi-stage random probability proportional to size sample (PPS) sampling method was utilized between 2013 and 2015, employing education level and proportion of floating population as stratification criteria for sampling purpose. The selection process for our sample frame relied upon data derived from the Sixth National Population Census.

#### Sampling procedure:.

The multi-stage sampling approach performed in four stages (Table 1).

**Table 1:** Multi-stage random PPS sample.

Stages	Sample units
First stage	Selected 28 counties
Second stage	Selected 4 schools in each selected counties
Third stage	Selected 4 classes in each selected schools
Fourth stage	Selected all students in the sample class

**County Selection**: In the first stage, 28 counties were selected based on average educational level and proportion of floating population.

**School Selection**: In the second stage, 112 schools within these counties were chosen.

**Class Selection**: In the third stage, classes from grade seven and grade nine were selected, totaling 483 classes.

**Student Selection**: In the final stage, a total of 20,000 students were included in the study.

This study utilized baseline and follow-up data to examine the association between sleep duration and academic performance, as well as to explore the mediating role of BMI in this relationship. The “clsids” number was used for data matching purposes. Following the research objective, missing data pertaining to the core variables were excluded, resulting in a final sample size of 7,218 students included in this observational analysis.

## Measurement

### Academic achievement

The academic performance of adolescent students was evaluated based on the scores obtained in the autumn midterm examinations for Chinese, mathematics, and English classes during 2013 and 2014 academic year, which were provided by the school administration. These scores were subsequently converted into a percentage system following previous research [22]. Specifically, this study focused on the calculating the average overall score of the autumn midterm examination for Chinese, mathematics, and English.

### Household register

The household register, commonly known as “huko” in Chinese, has been extensively employed for evaluating students residing in both rural and urban areas [23,24]. It was represented by dichotomous variables with the inquiry being “What is your huko?”. The response options include rural (coded as “1”) and urban (coded as “2”).

### Body mass index

The measurement of physical health was conducted using BMI, a conventional indicator for assessing individual’s weight status and judging physical fitness [19,25]. Students’ weight and height were collected from the baseline survey. The BMI was calculated using the formula:weight(kg)/height^2^(M). Based on *the National Student Physical Health Standard (version 2014)* [26], students’ BMI were categorized into four groups: underweight, normal weight, overweight, and obese.

### Sleep duration

The sleep duration was assessed and quantified in hours and minutes in this study. Participants were asked the following questions to ascertain their sleep time: “How many hours did you sleep last night?” or “What time did you usually go to bed and wake up during the past week?”. Participants provided their responses to the question based on their recent sleep duration or average time over the past week, aiming to mitigate potential recall bias. The sleep duration was calculated in 10-minute intervals for the purpose of explaining the results.

### Covariant

Parental background, social demographic characteristics of students, and students’ academic goal were controlled based on previous studies investigating students’ education performance [19,27,28]. Parental occupation was assessed by asking “What is your mother or father’s occupation?”, with responses coded from 1 to 14 according to occupational type. Parental education was assessed by inquiring “What is your mother or father’s highest level of education?”, with responses coded from 1 to 9 according to educational attainment. Parental requirement regarding their children’s academic performance were measured by inquiring “What are your parents requirement for your academic performance?”. Responses were categorized as follows: “top 5 in the class=1”, “above the average=2”, “average=3”, “no special requirement=4”. Educational expectations of parents were assessed by inquiring “What are your parents’ expectation for your education?”, with response coded from 1 to 10 based on desired educational outcomes. The question regarding whether the student is an only child or not was assessed by inquiring “Are you an only child?”, with responses coded as ‘yes=1’ and ‘no=2’. Sibling information was collected through the question “How many siblings do you have?”, with answers ranging from 0 to 12. Academic goals were assessed through the question “What are your academic goals?”, with responses coded from 1 to 10 according to desired educational outcomes. Future career plans were evaluated using the question “What are your future career plans?”, with responses coded from 1 to 10 according to work type. Family socioeconomic status was assessed using subjective self-evaluation, ranging from 1 (extreme poverty) to 5 (extreme wealth). Students were asked about their residing arrangement with the dichotomous response of whether you resided on-campus or off-campus.

### Data analysis

This study employed four main analytical strategies to investigate the association between sleep duration and academic performance among adolescent students, while also examined the mediating role of BMI after controlling for academic goals, future work planning, and demographic variables. Firstly, descriptive statistical analysis was conducted to test the basic information regarding students’ academic performance. Secondly, given that the dependent variable academic performance was a continuous variable, Ordinary Least Squares (OLS) regression was employed to examine the relationship between sleep duration and academic performance among adolescent students. Additionally, being consistent with previous study [29], subsample regression analysis was performed on rural-urban adolescent students to examine whether sleep duration have differential effects on academic performance among them. Finally, the Product of Coefficients Approach [30] was employed to evaluate the significance of BMI as mediator in this study. All data were analyzed using Stata (16.0SE), developed by Stata Corp., College Station, TX, USA. A significance level of P < 0.05 was considered statistically significant.

### Ethics statement

This research received ethical approval from the Ethical Review Board of Beijing Normal University and was conducted in accordance with the principal outlined in the Declaration of Helsinki.

## Results

### Basic characteristics

Table 2 presented the basic characteristics between rural and urban students, where the number, percentage of each variable were calculated. The results revealed significant disparities in family economy, parents’ education, parents’ occupation, siblings and BMI between rural and urban students. However, no substantial differences were observed regarding parents’ academic requirement and expectation, self-expectation and future career plan between rural and urban students.

**Table 2:** Basic characteristics of the observations.

		Total		Rural		Urban	
	Variables	N	Proportion	N	Proportion	N	Proportion
**Student characteristics**	**Gender**						
	Male	3,733	52.29%	2,011	53.71%	1,722	50.72%
	Female	3,406	47.71%	1,733	46.29%	16,738	49.28%
	**Self-expectation**						
	Middle school	151	2.13%	102	2.74%	49	1.45%
	High school	858	12.08%	583	15.66%	275	8.14%
	College and above	5,813	81.87%	2,885	77.49%	2,928	86.70%
	Indifferent	278	81.87%	153	4.11%	125	3.70%
	**Future work**						
	Government officer	623	8.74%	311	8.31%	312	9.20%
	Business manager	899	12.61%	440	11.76%	459	13.54%
	Professional	2,648	37.13%	1,357	36.27%	1,291	38.07%
	Art and athlete	1,051	14.74%	491	13.12%	560	16.51%
	Service industry	490	6.87%	342	9.14%	148	4.36%
	Soldier	554	7.77%	344	9.20%	210	6.19%
	Self-employed entrepreneurs	227	3.18%	134	3.58%	93	2.74%
	Others	215	3.01%	101	2.70%	114	3.36%
	Indifferent	425	5.96%	221	5.91%	204	6.02%
**Family characteristics**	**only child**						
	Yes	3,298	46.77%	1,034	28.06%	2,264	67.26%
	No	3,753	53.23%	2,651	71.94%	1,102	32.74%
	**Mother’s education**						
	Uneducated	217	3.10%	150	4.07%	67	2.02%
	Middle school and below	4,132	58.97%	2,492	67.55%	1,640	49.43%
	High school and technical secondary school	1,691	24.13%	775	21.01%	916	27.61%
	College and above	967	13.80%	272	7.37%	695	20.95%
	**Father’s education**						
	Uneducated	37	0.52%	23	0.62%	14	0.42%
	Middle school and below	3,835	54.82%	2,388	64.84%	1,447	43.69%
	High school and technical secondary school	1,941	27.75%	933	25.33%	1,008	30.43%
	College and above	1,182	16.90%	339	9.20%	843	25.45%
	**Mother’s work**						
	Government officer	367	2.58%	42	0.57%	325	4.74%
	Business manager	816	5.74%	142	1.93%	674	9.83%
	Professional	2,191	15.41%	794	10.79%	1,397	20.37%
	Servant	3,329	23.41%	1,379	18.73%	1,950	28.43%
	Technology worker	3,400	23.91%	2,453	33.32%	947	13.81%
	Farmer, herdsman, fisher	1,458	10.25%	1,112	15.10%	346	5.05%
	Junior class labor	287	2.02%	195	2.65%	92	1.34%
	Self-employed entrepreneur	1,130	7.95%	593	8.05%	537	7.83%
	Unemployment or retire	977	6.87%	509	6.91%	468	6.82%
	Others	265	1.86%	143	1.94%	122	1.78%
	**Father’s work**						
	Government officer	611	4.29%	77	1.04%	534	7.78%
	Business manager	1,355	9.51%	289	3.92%	1,066	15.52%
	Professional	2,927	20.55%	1,289	17.48%	1,638	23.85%
	Servant	2,309	16.21%	1,022	13.86%	1,287	18.74%
	Technology worker	3,890	27.31%	2,751	37.30%	1,139	16.59%
	Farmer, herdsman, fisher	1,326	9.31%	1,035	14.03%	291	4.24%
	Junior class labor	123	0.86%	62	0.84%	61	0.89%
	Self-employed entrepreneur	1,238	8.69%	653	8.85%	585	8.52%
	Unemployment or retire	194	1.36%	66	0.89%	128	1.86%
	Others	269	1.89%	131	1.78%	138	2.01%
	**Family income**						
	Low	1,814	12.90%	1,354	18.59%	460	6.79%
	Middle	10,416	74.09%	5,381	73.87%	5,035	74.33%
	Rich	1,828	13.00%	549	7.54%	1,279	18.88%
	**Parents’ requirement**						
	Top 5	3,884	27.02%	2,015	27.06%	1,869	26.98%
	Above average	6,973	48.51%	3,552	47.70%	3,421	49.38%
	Average	2,448	17.03%	1,298	17.43%	1,150	16.60%
	No requirement	1,070	7.44%	582	7.82%	488	7.04%
	**Parents’ expectation**						
	Middle school	223	1.55%	149	2.00%	74	1.07%
	High school or technical school	1,813	12.63%	1,198	16.10%	615	8.89%
	College and above	11,853	82.58%	5,843	78.55%	6,010	86.91%
	Indifferent	465	3.24%	249	3.35%	216	3.12%
**Health characteristics**	**BMI**						
	Underweight	1,428	9.89%	801	10.71%	627	9.01%
	Normal	11,116	77.00%	5,886	78.70%	5,230	75.18%
	Overweight	1,087	7.53%	447	5.98%	640	9.20%
	Obesity	805	5.58%	345	4.61%	460	6.61%
	**Sleep duration**						
	≤9h	9,743	67.49%	4,709	62.96%	5,034	72.36%
	>9h	4,693	32.51%	2,770	37.04%	1,923	27.64%

Table 3 presented the comparison results of sleep duration on students’ residing arrangement and BMI in both rural and urban area. The analysis of the full sample showed that students residing on-campus had longer sleep duration than those residing off-campus. Subgroup analysis indicated that urban students residing off-campus had the shortest sleep duration among the all groups, even shorter than overall average. In terms of BMI, underweight students exhibited a longer sleep duration than overweight and obese students in the full sample analysis. Subgroup analysis further demonstrated that overweight and obese urban students had the shortest sleep duration among all groups, even shorter than overall average.

**Table 3:** The differences of sleep duration on residing arrangement and BMI among rural-urban adolescent students.

Variables	Total (mean)	Rural (mean)	Urban (mean)
Sleep duration with residing on-campus	8.43h	8.45h	8.35h
Sleep duration with residing off-campus	8.07h	8.21h	7.98h
**BMI**			
Underweigt	8.30h	8.30h	8.10h
Normal	8.20h	8.20h	8h
Overweight	8h	8.20h	7.80h
Obese	8h	8.20h	7.80h
Observation	7,218	3,677	3,541

### The influence of sleep duration on rural-urban students’ academic performance

The results of the OLS regression analysis on the full sample revealed a significant negative association between sleep duration and academic performance among all students (Table 4). Specifically, an increase of 10-minute in sleep duration was found to be associated with a decrease of 6.3% in academic performance for rural-urban students. Subgroup analyses further demonstrated that a 10-minute increase in sleep duration led to an 8.5% decrease in academic performance for rural adolescent students, while urban students experienced a 6.7% decrease under similar circumstances.

**Table 4:** The influence of sleep duration on rural-urban students’ academic performance.

Variables	Total (β, 95%CI)	Std.Err.	Rural students (β, 95%CI)	Std.Err.	Urban students (β, 95%CI)	Std.Err.
Sleep duration	-0.063** (-0.107,-0.015)	0.024	-0.085** (-0.125,-0.152)	0.049	-0.067* (-0.139,0.032)	0.044
BMI	-0.117** (-0.190,-0.033)	0.039	-0.146** (-0.191,0.005)	0.450	-0.214*** (-0.268,-0.003)	0.068
Gender	-0.132 (-0.069,-0.412)	0.281	0.045 (-0.607,0.704)	0.334	-0.406 (0.458,1.234)	0.525
Self-expectation	0.563*** (1.102,2.314)	0.309	0.455*** (0.219,0.695)	0.121	0.846*** (-0.286,0.023)	0.198
Future work	-0.188*** (-0.284,-0.115)	0.043	-0.219*** (-0.321,-0.117)	0.052	-0.131^+^ (-1.106,2.951)	0.079
Only child	1.307^+^ (-0.044,2.795)	0.724	1.552 (-0.619,3.684)	1.097	0.922 (-1.106,2.951)	1.033
Number of siblings	0.327 (-0.084,0.764)	0.216	0.384 (-0.093,0.880)	0.248	0.263 (-0.560,1.125)	0.439
Mother’s work	0.098^+^ (-0.053,0.243)	0.076	0.107 (-0.043,0.259)	0.077	0.081 (-0.082,0.244)	0.083
Father’s work	0.115* (0.012,0.322)	0.079	0.040 (-0.105,0.183)	0.073	0.169* (0.018,0.321)	0.077
Family economy	-0.568* (-1.075,-0.168)	0.231	-0.155 (0.671,0.387)	0.270	-1.675*** (-2.563,-0.787)	0.452
Parents’ demand	-3.739*** (-4.122,-3.436)	0.175	-3.711*** (-4.128,-3.306)	0.209	-3.723*** (-4.356,-3.090)	0.322
Parents’ expectation	0.635*** (0.539,0.936)	0.101	0.655*** (0.399,0.904)	0.129	0.601** (0.178,1.025)	0.216
Residing on-campus or off-campus	-0.101 (-0.719,0.457)	0.299	-0.021 (-0.697,0.634)	0.340	-0.396 (-1.636,0.845)	0.632
Mother’s education	-0.135 (-0.861,0.157)	0.260	-0.054 (-0.292,0.182)	0.121	-0.250 (-0.584,0.084)	0.170
Father’s education	0.106 (0.297,0.715)	0.259	0.126 (-0.108,0.357)	0.119	0.125 (-0.204,0.455)	0.168
R-squared	0.248		0.249		0.259	
Observations	7,218		3,677		3,541	

Standard errors in parentheses *** p < 0.001, ** p < 0.01, * p < 0.05, + p < 0.1.

### The influence of sleep duration on academic performance of students’ residing both on-campus and off-campus

Considering the relationship between sleep duration and residing arrangement, an interaction analysis was conducted to examine the impact of sleep duration and residing arrangement (on-campus or off-campus). The full sample analysis presented that students’ academic performance was significant influenced by their residing arrangement (Table 5). However, subgroup analysis presented that this influence was only significant among rural students, while no significant effect observed among urban students (Table 5). Specifically, an increase of 10-minute in sleep duration resulted in a significant decline of 50% in academic performance for rural students residing off-campus, and a corresponding decrease of 43% for residing on-campus.

**Table 5:** The interaction results of residing arrangement and sleep duration among rural-urban adolescent students.

Variables	Total (β, 95%CI)	Std.Err.	Rural students (β, 95%CI)	Std.Err.	Urban students (β, 95%CI)	Std.Err.
Residing arrangement × sleep						
1.yes	-0.059** (-0.697,-0.251)	0.023	-0.433*** (-0.619,-0.067)	0.127	-0.197 (-0.588,0.254),	0.199
2.no	-0.062*** (-0.745,-0.285)	0.024	-0.503*** (0.635,0.063)	0.131	-0.284 (-0.639,0.208)	0.186
Observation number	7,218		3,677		3,541	
R-squared	0.248		0.007		0.006	
Akaike crit. (AIC)	46582.989		24591.843		12748.846	
Bayesian crit. (BIC)	46610.058		24616.355		12770.754	

** p < 0.01, * p < 0.05, + p < 0.1.

### The BMI mediated sleep duration and academic performance among rural-urban adolescent students

**Fig 1:**
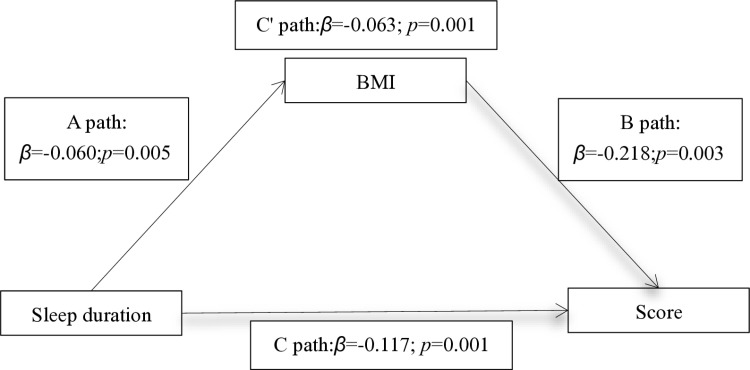
The transmission mechanism between the relationship of sleep duration and students’ academic performance. BMI served as a mediator between sleep duration and their academic scores. Note: Path A: the IV (sleep duration) significant influenced the Mediator (BMI). Path B: the mediator (BMI) significantly influenced DV (academic performance). Path C: the IV (sleep duration) directly influenced the DV (academic performance). Path C′: the IV (sleep duration) partially influenced the DV (academic performance) on the addition of the mediator (BMI).

The Product of Coefficients Approach indicated that an indirect association existed between sleep duration of students and their academic performance after controlling for the covariant. The results in Table 6 and Fig 1 showed a significant influence (*p* < 0.001) of students’ sleep duration and their academic performance through BMI. (1) Path A showed that the adolescent students’ sleep duration had a significant influence on their BMI (*β* = -0.060, *p* < 0.01). (2) Path B showed the direct effect of BMI on academic performance among adolescent students (*β* = -0.218, *p* < 0.01). (3) Path C showed the direct effect of sleep duration on academic performance among adolescent students (*β* = -0.117, *p* < 0.001). (4) Path C’ showed the coeffect of sleep duration and BMI on academic performance among adolescent students (*β* = -0.063, *p* < 0.001). These results provide confirmation for the hypothesis that BMI serves as a mediator in the relationship between sleep duration and academic performance among adolescent students.

**Table 6:** The BMI mediated the sleep duration and the academic performance.

	A path (β)	B path (β)	C path (β)	C′ path (β)
	Direct effects of sleep duration on BMI	Direct effects of BMI on academic performance	Direct effects of sleep duration on academic performance	Coeffect of sleep duration and BMI
sleep duration	-0.060**		-0.117***	0.063***
BMI		-0.218**		
*cons*	5.662			8.551
*95% CI*	(-0.042,-0.014)	(-0.195,-0.038)	(-0.107, -0.014)	(-0.109,-0.038)
*Adjust-R* ^*2*^	0.276	0.258	0.246	0.256
	Medication effect	11.18%	Mediation effect of total effect	

Note: (1) *** p < 0.001, ** p < 0.01, * p < 0.05, + p < 0.1.

(2) Mediation effect calculation:*E* = *β*(A path) *β(B path)=(-0.060)*(-0.218)=0.013.

(3) Mediation effect size: *E* = *β*(A path) *β(B path) /*β*(C path)=(-0.060)*(-0.218)/0.117*100% = 11.18%.

(4) Mediation effect of total effect: *P* = E(Mediation effect)/E(Mediation effect) +*β*(C path)=11.18%/(11.18% + 11.7%)= 48.86%.

## Discussion

In this study, we explored the association between sleep duration and academic performance among adolescent students, and further explored its transmission mechanism. To our knowledge, this is the first study conducted in China to examine this association and explore its mechanism. The findings revealed a negatively influence of sleep duration on students’ academic performance, and the mediating analysis indicated that this influence was mediated by students’ BMI.

Recent studies have consistently demonstrated a positive association between increased sleep duration and students’ academic performance [11,31]. Zahra Ahmadi and Shabnam Omidvar testified that students with regular sleep patterns (8 hours of sleep per night) tended to achieve higher GPA scores compared to those with shorter nighttime sleep duration [32]. However, this study unveiled a negative relationship between increased sleep duration and students’ academic performance. That is, shorter sleep duration among adolescent students contributed to improve their academic performance. This can be attributed to the reallocation of sleep time towards studying, enabling students to consolidate their learning and prepare for acquiring new knowledge. Further subgroup analysis explained that this influence was greater among rural students compared to urban students. Previous study illustrated that urban students allocated their sleep time towards tutoring class, which aids in enhancing their academic performance [33]. Conversely, rural students exhibited longer sleep duration due to the absence of such tutoring class.

Interaction analysis revealed that the association of sleep duration and rural-urban students’ academic performance was moderated by students’ residing arrangement, indicating that students residing on-campus had a positive influence on academic performance compared to residing off-campus. This can be attributed to the fact that students residing on-campus adhered more strictly to regular sleep, ensuring dedicated study time. A recent study provide evidence supporting this result, as boarding schools enforced strict sleep schedules which students diligently followed to optimize their study time [34]. Further subgroup analysis explained that this interaction effect was significant among rural students, but not among urban students. The reason for this is that residing on-campus enables rural students to save travel time and ensure ample study hours. Additionally, the school strictly enforces schedules for further prioritize study time. Conversely, rural students residing off-campus spent considerable time commuting or engaged in household chores, thereby reducing their available study time hours [35]. In contrast, the results of interaction effect was not significant in urban students may attributed to the abundance of education resource (such as tutoring class) and higher education quality offered by urban schools, diminishing the role of residing arrangement in this association.

The key finding of this study was that BMI mediated the relationship between sleep duration and academic performance among adolescent students. Previous studies have confirmed the influence of sleep on students’ body weight [11,28,36]. Xunqiang Wang testified that insufficient sleep increased the risk of obesity [37]. Additionally, Philippa J Carter confirmed that insufficient sleep in adolescents increased the likelihood of developing overweight [38]. Another study reconfirmed that adolescents who slept less than 8 hours per night had an increased risk of overweight and obesity [39]. Once again, the results of this study explained the profound association between sleep duration and body mass index (BMI). The controversial evidence of previous studies has also established the association between being overweight or obese and iron deficiency anemia pathogenesis [40,41], which detrimentally affects adolescent brain development [42]. Similarly, another study provided the evidence that overweight and obese adolescents were more likely to experience feelings of inferiority, anxiety-related issues, social behavior problems (such as introverted personality traits, suspicion, aggression tendencies), all influencing their academic performance [43]. Previous studies have also confirmed that underweight adolescents were more prone to experiencing malnutrition leading to hypodevelopment of central nervous system [44,45]. The evidence from previous study has confirmed that the central nervous plays a critical role in psychomotor development, intelligence development, and language development, which were essential for their academic performance [46].

Findings from this study contribute to the growing body of evidence regarding the involvement sleep duration and BMI on academic performance in students at all educational stages. As highlighted above, shorter sleep duration was found to be conductive to academic performance of adolescent students. However, it is important to note that this seemingly positive academic performance comes at the cost of compromised physical health, including increased risk for overweight and obesity [36]. In accordance with the policy of *Management standards for compulsory education schools,* the recommended minimum sleep duration for adolescent students should not be less than 9 hours*.* The results of this study confirmed that China’s rural-urban adolescent students were obtaining less than 8.5 hours of sleep per night, with some even getting less than 8 hours.

## Limitations

There are certain limitations in this study that must be acknowledged. Firstly, this study primarily centered on controlling for family background and health-related variables, thereby neglecting other potential factors such as psychological and cognitive variables that may exert influence on academic achievement. Secondly, this study used two waves of data to analyze the association, which cannot be employed to analyze the causal relationship between sleep duration and academic performance. Future research should consider employing longitudinal studies to address this limitation and establish causality. Thirdly, while analyzing the mechanism of sleep duration and academic performance in this study, it was challenging to resolve the endogenous problem. Future research should incorporate instrumental variables tackle the issue of endogeneity.

### Practical implications

The findings of this study are expected to stimulate a substantial number of researchers to investigate the correlation between health-related factors and students’ academic performance in China. Additionally, these results will capture the attention of the Chinese government concerning students’ physical health and their academic performance, potentially leading to the formulation of policy interventions aimed at promoting adequate sleep duration for students and alleviating their academic burden. Most significantly, this study’s outcomes have the potential to revolutionize perspectives on educational management and public opinion.

## Conclusion

In conclusion, this study was the first to identify a predictive association between sleep duration and academic performance, as well as its mechanism, among rural-urban adolescent students. Sleep duration significantly influenced academic performance in adolescent students, with this influence being moderated by residing arrangement and mediated by the BMI. Subgroup analysis further revealed that this influence differs in rural and urban adolescent students. The findings of this study highlighted the importance for government and education departments to prioritize students’ physical health alongside academic scores. Therefore, screening for sleep duration may be beneficial in promoting overall wellness among adolescent students. We propose that the education department should undertake a comprehensive reform of the educational system, shifting focus from prioritizing grades to placing greater emphasis on safeguarding the well-being of adolescent students. Future studies may further explore the intervention methods for improving sleep quality and ensuring sufficient rest among adolescent students.

## Abbreviations

BMI: body mass index
CEPS: China education panel survey
PPS: probability proportional to size sample
OLS: ordinary least squares
SEM: structure equation model
